# Early treatment of Atrial fibrillation for Stroke prevention Trial in acute STROKE (EAST-STROKE): protocol for an international investigator-initiated, prospective, randomised, open, blinded endpoint assessment (PROBE) interventional multi-centre trial

**DOI:** 10.1093/esj/aakag022

**Published:** 2026-05-19

**Authors:** Märit Jensen, Antonia Zapf, Anika Buchholz, Annika Möhl, Jason G Andrade, Alan Cameron, Bruce C V Campbell, Urs Fischer, Michael D Hill, Peter Kelly, Jonathan M Kalman, Peter Loh, Carlos Molina, Eduard Guasch, Andreas Metzner, G André Ng, Jorge Pagola, Octávio M Pontes-Neto, Tobias Reichlin, Rustam Al-Shahi Salman, Ashkan Shoamanesh, Luciano A Sposato, H Bart van der Worp, Paulus Kirchhof, Götz Thomalla

**Affiliations:** Department of Neurology, University Medical Center Hamburg-Eppendorf, Hamburg 20246, Germany; German Centre for Cardiovascular Research (DZHK e.V.), Hamburg/Kiel/Lübeck, Germany; Institute of Medical Biometry and Epidemiology, University Medical Center Hamburg-Eppendorf, Hamburg, Germany; Institute of Medical Biometry and Epidemiology, University Medical Center Hamburg-Eppendorf, Hamburg, Germany; Institute of Medical Biometry and Epidemiology, University Medical Center Hamburg-Eppendorf, Hamburg, Germany; Vancouver General Hospital, Vancouver, British Columbia, Canada; Montréal Heart Institute, Université de Montréal, Montréal, Québec, Canada; Center for Cardiovascular Innovation, Vancouver, British Columbia, Canada; School of Cardiovascular and Metabolic Health, University of Glasgow, Glasgow, United Kingdom; Department of Medicine and Neurology, Melbourne Brain Centre at the Royal Melbourne Hospital, University of Melbourne, Parkville, Victoria, Australia; The Florey Institute of Neuroscience and Mental Health, University of Melbourne, Parkville, Victoria, Australia; Department of Neurology, University of Bern, Bern, Switzerland; Department of Clinical Neurosciences, Hotchkiss Brain Institute, Cumming School of Medicine, University of Calgary, Calgary, Alberta, Canada; Stroke Clinical Trials Network Ireland and School of Medicine, University College Dublin, Dublin, Ireland; Department of Cardiology, The Royal Melbourne Hospital, Melbourne, Australia; Department of Cardiology, University Medical Center Utrecht, Heidelberglaan 100, Utrecht, The Netherlands; Stroke Unit, Neurology Department, Vall d'Hebron University Hospital, Barcelona, Spain; Cardiology Department, Hospital Clinic Barcelona, Barcelona, Catalonia, Spain; Institut d’Investigacions Biomèdiques August Pi i Sunyer (IDIBAPS), Barcelona, Catalonia, Spain; Medicine Department, Universitat de Barcelona, Barcelona, Catalonia, Spain; German Centre for Cardiovascular Research (DZHK e.V.), Hamburg/Kiel/Lübeck, Germany; Department of Cardiology, University Heart and Vascular Center Hamburg, University Medical Center Hamburg-Eppendorf, Hamburg 20246, Germany; Division of Cardiovascular Sciences, British Heart Foundation Leicester Centre of Research Excellence, University of Leicester, National Institute for Health Research Leicester Biomedical Research Centre, Glenfield Hospital, Leicester, United Kingdom; Stroke Unit, Neurology Department, Vall d'Hebron University Hospital, Barcelona, Spain; Stroke Service, Neurology Division, Ribeirão Preto Medical School, University of São Paulo, Ribeirão Preto, Brazil; Department of Cardiology, Inselspital Bern University Hospital, University of Bern, Bern, Switzerland; Institute for Neuroscience and Cardiovascular Research, The University of Edinburgh, Edinburgh, United Kingdom; Division of Neurology, Department of Medicine, Population Health Research Institute, McMaster University, Hamilton, Ontario, Canada; Department of Clinical Neurological Sciences, Schulich School of Medicine and Dentistry, Western University, London, Ontario, Canada; Heart & Brain Laboratory, Western University, London, Ontario, Canada; Department of Neurology and Neurosurgery, Brain Center, University Medical Center Utrecht, Utrecht, The Netherlands; German Centre for Cardiovascular Research (DZHK e.V.), Hamburg/Kiel/Lübeck, Germany; Department of Cardiology, University Heart and Vascular Center Hamburg, University Medical Center Hamburg-Eppendorf, Hamburg 20246, Germany; Institute of Cardiovascular Sciences, University of Birmingham, Birmingham, United Kingdom; Department of Neurology, University Medical Center Hamburg-Eppendorf, Hamburg 20246, Germany

**Keywords:** acute ischaemic stroke, atrial fibrillation, rhythm-control therapy, secondary stroke prevention

## Abstract

**Background:**

Acute ischaemic stroke patients with atrial fibrillation (AF) are at high risk of suffering a recurrent stroke or other cardiovascular complications. It is uncertain whether early rhythm-control therapy is effective and safe in preventing recurrent strokes and cardiovascular complications in these patients.

**Study design:**

Early treatment of Atrial fibrillation for Stroke prevention Trial in acute STROKE (EAST-STROKE) is an international investigator-initiated, prospective, randomised, open, blinded endpoint assessment (PROBE) interventional multi-centre trial. Patients with acute ischaemic stroke and AF will be randomised within 4 weeks of stroke (1:1) to receive either early rhythm control and usual care or usual care alone. Usual care includes oral anticoagulation, rate control and treatment of cardiovascular conditions. Early rhythm control additionally comprises treatment with antiarrhythmic drugs, AF ablation or cardioversion. A minimum of 1746 participants will be randomised to observe 351 events. The adaptive design includes one interim analysis with sample size re-estimation after 50% of events.

**Study endpoints:**

The primary outcome is a composite of first recurrent ischaemic stroke, haemorrhagic stroke, unclassified stroke, cardiovascular death or hospitalisation due to worsening of heart failure or due to acute coronary syndrome, analysed as time to the first occurrence. Secondary outcomes include individual components of the primary outcome, functional status and patient-reported outcome measures. Safety outcomes comprise all-cause mortality and adverse events. Patients will be followed-up until the end of the trial with a minimum follow-up period of 24 months and an expected mean follow-up period of 42 months.

**Summary:**

EAST-STROKE will determine whether early rhythm-control therapy in addition to usual care is effective and safe in patients with acute ischaemic stroke and AF.

**Trial registration:**

ClinicalTrials.gov Identifier: NCT05293080; EUCT-No.: 2025-521260-35-00.

## Graphical Abstract

**Graphical Abstract:**
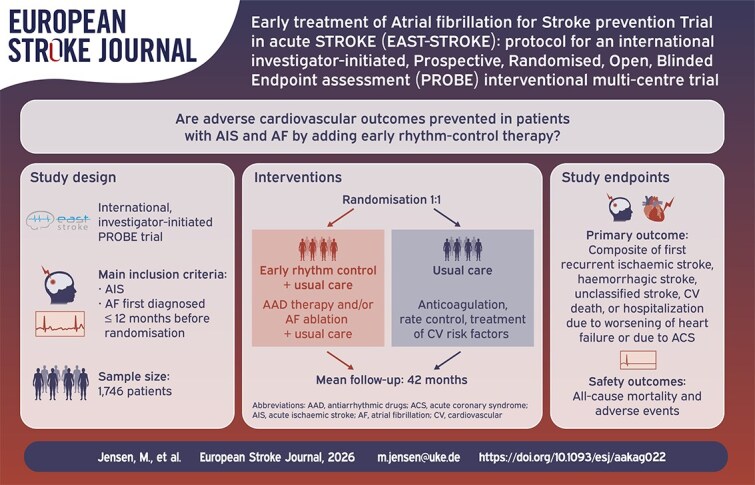

## Introduction and rationale

Recurrent stroke is a major public health concern, affecting up to 25% of patients with ischaemic stroke within 5 years.^1^ Atrial fibrillation (AF) significantly increases stroke risk despite oral anticoagulation (OAC), leaving patients vulnerable to recurrent, often severe strokes.^2,3^ Moreover, AF is also associated with an increased risk of other major cardiovascular events such as myocardial infarction and heart failure.^4–7^

Historically, the risk of stroke was thought to be similar regardless of AF burden, and secondary stroke prevention focused not on AF itself but rather on preventing AF-associated thromboembolism via OAC. However, recent data point towards the role of AF burden, with lower stroke risk in patients with paroxysmal AF compared to chronic AF.^8^ Previous rhythm-control trials failed to show a stroke prevention benefit, likely due to patient selection, suboptimal rhythm-control rates and discontinuation of anticoagulation after restoration of sinus rhythm. The more recent Early Treatment of Atrial Fibrillation for Stroke Prevention Trial (EAST-AFNET 4) demonstrated that early rhythm control reduces stroke, cardiovascular death and hospitalisations in patients with AF and increased cardiovascular risk.^9^

The efficacy and safety of early rhythm control in patients with AF and acute ischaemic stroke have not yet been evaluated in an adequately powered outcome trial. The RAFAS (Risk and benefits of urgent rhythm control of Atrial Fibrillation in patients with acute Stroke) trial, which randomised 300 patients with acute ischaemic stroke and AF, suggested a reduction in recurrent stroke at 12 months with early rhythm-control therapy. However, its findings are limited by the small sample size and low number of events.^10^ A subgroup analysis from the EAST-AFNET 4 trial showed a strong effect of early rhythm control in patients with a history of stroke compared to those without, but EAST-AFNET 4 did not include patients with acute stroke. Large observational studies further support the potential benefit of early rhythm control in patients with AF and ischaemic stroke.^11^

Despite these findings, substantial uncertainty remains, as rhythm-control therapy carries potential risks that may be aggravated after an acute stroke, particularly in elderly and frail patients, highlighting the need for systematic safety evaluation before broader clinical adoption. An international survey underscored this clinical equipoise, with 80% of centres rarely using rhythm-control therapy in patients with acute ischaemic stroke and AF and 90% expressing willingness to enroll patients in a randomised trial.^12^ These findings were corroborated by a Canadian survey, in which only a minority of respondents reported offering treatments to restore sinus rhythm in patients with acute stroke and AF, whereas the vast majority (84%) indicated they would be willing to randomise patients in a clinical trial of early rhythm control after acute ischaemic stroke.^13,14^

Although rhythm-control therapies are associated with recognised risks, these are well characterised and often manageable. In EAST-AFNET 4, over a median follow-up of 5.1 years per patient, serious adverse events related to rhythm-control therapy occurred in 4.9% of patients assigned to early rhythm control.^9^ In particular, ventricular proarrhythmia with antiarrhythmic drugs can often be avoided by appropriate patient selection and electrocardiogram (ECG) monitoring.

Taken together, existing evidence suggests that early rhythm-control therapy may provide meaningful clinical benefit with acceptable safety. Therefore, the Early treatment of Atrial fibrillation for Stroke prevention Trial in acute STROKE (EAST-STROKE) aims to evaluate the efficacy and safety of early rhythm control in preventing recurrent stroke and major cardiovascular events in patients with AF and acute ischaemic stroke. Secondary objectives include assessing health-related quality of life, functional outcomes and cost-effectiveness.

## Methods

### Study design

EAST-STROKE is an international investigator-initiated, prospective, randomised (1:1), open, blinded endpoint assessment (PROBE) parallel-group interventional multi-centre trial to test whether an early, comprehensive, rhythm-control therapy in addition to usual care can reduce the rate of adverse cardiovascular outcomes in patients with acute ischaemic stroke and AF compared to usual care alone. Because it is addressing a novel process of care, it is considered a comparative effectiveness trial. The trial will be conducted in study centres in Europe, Australia and Canada. Study centres in countries of other continents may follow.

### Participant population

EAST-STROKE will randomise a minimum of 1746 patients with acute ischaemic stroke and AF. Inclusion and exclusion criteria are listed in Table 1. EAST-STROKE aims to achieve a balanced sex distribution in enrolment.

**Table 1:** Inclusion and exclusion criteria.

**Inclusion criteria** Acute ischaemic stroke confirmed by brain imaging or clinical diagnosis Randomisation within 4 weeks after stroke (patients are expected to be randomised as soon as possible after stroke, most often during initial hospitalisation for acute stroke treatment) AF first detected ≤ 1 year before randomisation (also paroxysmal AF) At least 1 ECG within recent 12 months that documents AF (documented current AF during index hospitalisation OR documented AF within the previous 12 months with sinus rhythm at the time of screening). The diagnosis of AF requires documentation by ECG. This will typically be a 6-lead or 12-lead ECG recorded in a medical practice or hospital. Documentation by consumer electronics (smart watches, apps or patient-operated ECG cards) is not sufficient as qualifying ECGs. Documentation on inpatient ECG-monitoring or a Holter ECG is acceptable as long as the requirements for AF duration outlined by current AF guidelines are met Age > 45 years Provision of written informed consent by the patient or—in case of incapacity of the patient—by a proxy according to national or local regulation **Exclusion criteria** *General exclusion criteria* End-stage cancer or life-expectancy < 12 months Participation in another clinical intervention trial, either within the past 2 months or ongoing Previous participation in the EAST-STROKE trial Pregnant women, women of childbearing potential not on adequate birth control (ie, highly effective methods of contraception with failure rate < 1%), breastfeeding women; for the purpose of this trial, a woman is considered of childbearing potential, ie, fertile, following menarche and until becoming post-menopausal unless permanently sterile (permanent sterilisation methods include hysterectomy, bilateral salpingectomy and bilateral oophorectomy; a post-menopausal state is defined as no menses for 12 months without an alternative medical cause; if a female participant cannot definitively attest to the absence of reproductive potential, a negative urine or serum ß-hCG test must be performed prior to recruitment) Drug abuse *Exclusion criteria related to acute ischaemic stroke* Severe stroke (ie, bedridden and fully dependent patients, mRS > 4) Any severe neurological complications after stroke that might impede the study results (eg, symptomatic ICH) *Exclusion criteria related to a cardiac condition* History of AF first diagnosed > 12 months prior to randomisation Patients not suitable for rhythm control of AF Prior AF ablation or surgical therapy for AF Severe mitral valve stenosis Prosthetic mitral valve Left atrial thrombi *Exclusion criteria based on laboratory abnormalities* Clinically relevant hepatic dysfunction requiring specific therapy Clinically manifest thyroid dysfunction requiring therapy. After successful treatment of thyroid dysfunction, patients may be enrolled when their thyroid function is controlled Severe renal impairment (stage IV or V; creatinine clearance < 30 mL/min, or requiring or almost requiring dialysis) *Exclusion criteria for specific investigational medicinal products* All contraindications listed in the summary of product characteristics (SmPC) of the specific investigational medicinal products considered for treatment in both treatment arms (intervention and comparator) apply and must be reviewed by the investigators prior to initiating any specific medical treatment

Abbreviations: AF, atrial fibrillation; EAST-STROKE, Early treatment of Atrial fibrillation for Stroke prevention Trial in acute STROKE; ECG: electrocardiogram; hCG, human chorionic gonadotropin; ICH, intracranial haemorrhage.

### Randomisation and blinding

Eligible patients will be randomised 1:1 to usual care (comparator) or usual care and early rhythm control (intervention). Randomisation will be stratified by country, age group (<70 years vs ≥70 years) and NIHSS score (<8 vs ≥8) using block randomisation with varying block sizes. Patients should be randomised as early as possible and must be randomised within 4 weeks of the index stroke onset date, preferably during hospitalisation.

Assessment of outcome events will be performed by assessors who are not aware of randomised group allocation. In addition, a blinded Endpoint Adjudication Committee will adjudicate all primary outcome events including all events that result in a hospitalisation.

### Treatment

EAST-STROKE is a comparative effectiveness trial that compares 2 treatment strategies in patients with acute ischaemic stroke and AF: usual care alone versus usual care and early rhythm control. All treatments (pharmacotherapy, cardioversion, ablation therapy) follow current standards and local practice with approved drugs and devices; no unapproved treatments are used. Individual treatment decisions, to achieve the randomised treatment aims, will be made by site teams.

The control arm follows standard AF management, which includes OAC, rate control and therapy of cardiovascular conditions in line with national and international guidelines. Anticoagulation therapy typically includes direct oral anticoagulants, vitamin K antagonists or other approved agents, eg, low-molecular-weight heparin, with initiation and monitoring following local protocols. Rate control, a primary approach in usual care, involves beta-blockers, calcium channel antagonists or digitalis glycosides. Dosing is adjusted to maintain a resting heart rate within a clinically acceptable range, typically below 100 or 110 beats per minute, with close monitoring for bradycardia or AF-related cardiomyopathy. Cardiovascular comorbidities will be managed according to standard practice without differences between randomised groups.^15^ Rhythm-control therapy in the usual care group follows local practice and is typically reserved for patients with persistent symptoms despite rate control.

**Figure 1:**
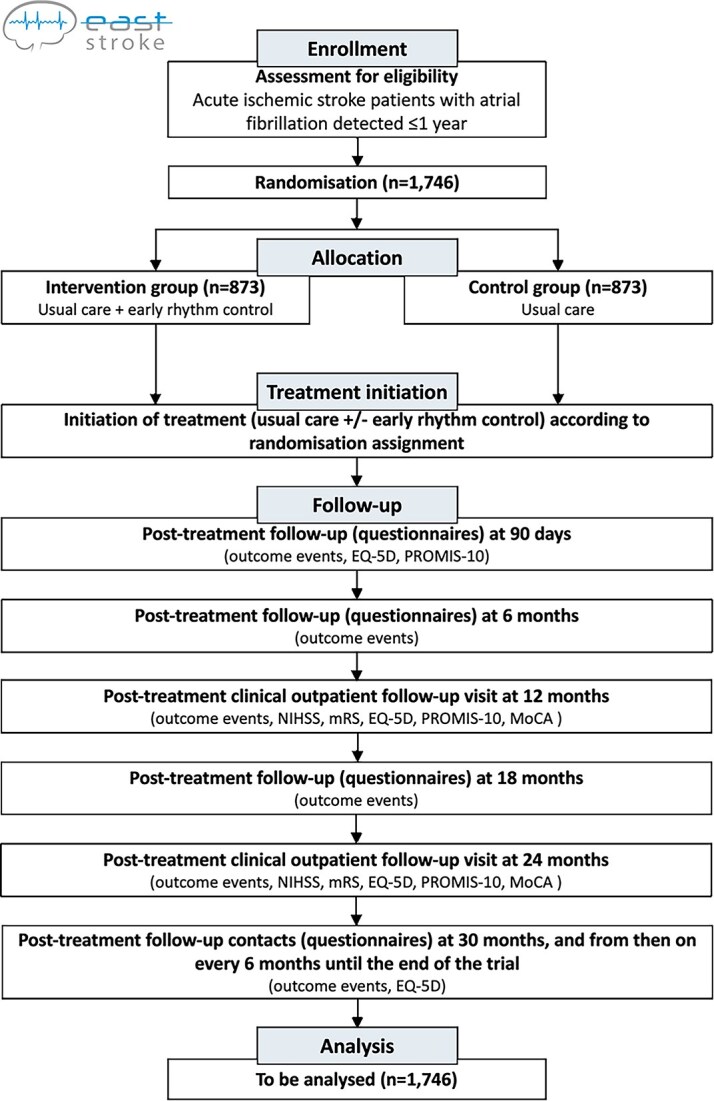
Trial schedule.

Patients in the intervention arm will receive early rhythm control as soon as possible after randomisation. Rhythm-control therapy includes antiarrhythmic drugs (mainly amiodarone, dronedarone, flecainide, propafenone), cardioversion, ablation or any combination of these 3 approaches, following local practice. We expect that antiarrhythmic drugs will be the primary means of starting early rhythm-control therapy, selected based on safety and standard practice. For long-term rhythm control, antiarrhythmic drugs, AF ablation or both can be used. When performed, AF ablation aims at pulmonary vein isolation following standard recommendations. Patients randomised to early rhythm control should be initiated on antiarrhythmic drug therapy within 2 weeks after randomisation or undergo AF ablation within 8 weeks after randomisation. Patients unable to receive rhythm control safely within this period are not eligible. After randomisation, treatment continues as part of standard care, with follow-up conducted per protocol (Figure 1).

### Clinical evaluation and follow-up

Clinical evaluation at baseline will include collection of demographic data and information on medical history and medication, determination of pre-stroke functional status, recording of stroke characteristics (eg, time of symptom onset, location of stroke), physical examination, assessment of the neurological deficit using the NIHSS, assessment of the functional status using the mRS, assessment of cognitive function using the Montreal Cognitive Assessment (MoCA), vital parameters (ie, systolic and diastolic blood pressure, heart rate, body temperature), 12-lead ECG, standard laboratory parameters (ie, screening routine laboratory tests by the local laboratory), pregnancy test if applicable and transthoracic echocardiography (TTE).

The follow-up period comprises follow-up visits performed by telephone call, and 2 on-site follow-up visits (see Figure 1). Follow-up starts at 90 (±10) days after randomisation. Further follow-up visits will be scheduled at 6 (±1) months, 18 (±1) months, 30 (±1) months, and from then on every 6 (±1) months until the end of the trial. On-site follow-up visits will be scheduled 12 (±1) and 24 (±1) months after randomisation.

During follow-up via telephone patients will be asked for any events that may have occurred since the last follow-up visit, including outcome events and adverse events. Adherence to study treatment and concomitant medication will be recorded at each follow-up. The follow-up at 90 days will include assessment of quality of life using the EuroQol 5-dimensional questionnaire (EQ-5D-5L) and Patient-Reported Outcomes Measurement Information System-10 (PROMIS-10). On-site follow-up visits will be performed 12 (±1) and 24 (±1) months after randomisation and will comprise physical examination, assessment of the neurological deficit using the NIHSS, assessment of the functional status using the mRS, assessment of cognitive function using the MoCA, assessment of quality of life using the EQ-5D-5L and PROMIS-10, vital parameters, 12-lead ECG, information on other treatment for AF (ie, anticoagulation, rate control), assessment of outcome events, assessment of adverse events and recording of study treatments and concomitant medication. At 24 months, a TTE will be performed.

### Primary outcome

The primary outcome is a composite of first recurrent ischaemic stroke, haemorrhagic stroke, unclassified stroke, cardiovascular death or hospitalisation due to worsening of heart failure or due to acute coronary syndrome, analysed as time to the first occurrence of one of the aforementioned components. Definitions of outcome components follow definitions developed by the FDA and others for cardiovascular trials.^16^

### Secondary outcomes

Secondary outcomes are the individual components of the primary outcome, recurrent disabling stroke, recurrent AF, cardiac rhythm (sinus rhythm vs AF) at 12 and 24 months, unplanned cardiovascular hospitalisation, number of cardiovascular hospitalisations, change from baseline in left ventricular ejection fraction at 24 months, mRS at 12 and 24 months, quality of life assessed with the EQ-5D and PROMIS-10 global health including domains on physical and mental health at 12 and 24 months, cognitive function assessed with MoCA at 12 and 24 months and cost of therapy.

### Safety outcomes

Assessment of safety will comprise all-cause mortality and adverse events with a special focus on serious adverse events related to the study intervention with special emphasis on proarrhythmia and complications due to interventions, and severe bleeding complications, ie, ICH and major bleeding.

### Overall duration of the trial

EAST-STROKE is an event-driven trial, ie, the trial will be terminated after the calculated number of required primary outcomes have occurred. Based on the initial sample size calculation 351 events are required. Yet this number may be increased based on the sample size re-calculation at the interim analysis. A duration of the entire trial of around 5 years is expected. Enrolment is expected to last about 36 months. All patients will be followed-up until the end of the trial with a minimum follow-up period of 24 months and an expected mean follow-up period of 42 months.

### Data safety monitoring board (DSMB)

An independent data safety monitoring board (DSMB) is monitoring the trial. The DSMB will meet at least annually and after the interim analysis, which will be performed after 50% of the planned number of events have occurred. In addition, the DSMB will meet ad hoc whenever safety relevant data occur that might have an influence on the study.

### Sample size calculation

The sample size calculation is based on an adaptive design with one interim analysis (O’Brien and Fleming type α-spending function) including a sample size re-estimation after 50% of the planned number of events have occurred, with overall 1-sided significance level for superiority of 2.5% and power 90%. An event rate (cumulative incidence) at 2 years of 15% and 10.8% is assumed in the usual care and usual care + early rhythm control group, respectively, reflecting a treatment effect with an expected cause-specific hazard ratio of 0.7. In addition, competing risks are taken into account (with expected rate of competing events of 1% and 2% in the usual care and early rhythm-control therapy group, respectively, based on results of an EAST-AFNET 4 subgroup analysis^17^) as well as a loss-to-follow-up of 5% of the total observation time. Based on these assumptions, a total of 1746 (873 per group) patients are initially planned to be enrolled to observe the required number of 351 events. To avoid an unmanageably large sample size, the sample size after recalculation will be restricted to a maximum of 2620 patients (chosen based on practical reasons including feasibility of enrolment and a clinically meaningful effect). Under the same assumptions used in the presented sample size calculation, this sample size limit would allow to detect a hazard ratio of about 0.756 (or larger effects) with 90% power.

### Statistical analysis

The primary analysis will follow the intention-to-treat principle in the full analysis set that includes all randomised patients as belonging to their randomised arm, regardless of the treatment received or whether protocol violations occurred. The test on group difference regarding the time to the primary outcome will be performed based on a Cox regression model including the treatment group, age, stroke severity and country. To account for the adaptive design of the trial, the inverse normal method (with weights of $\sqrt{0.5}$ for stages 1 and 2, respectively) is applied to the Cox regression analyses on stage-wise data^18^ at overall 1-sided significance level of 2.5% using O’Brien and Fleming type α-spending function. Safety analyses will be performed for all patients for whom one of the treatments was started. Rates of adverse events will be calculated by treatment group, according to type of event and system organ class. Secondary endpoints will be analysed descriptively, using regression models as appropriate for the respective type of data. Treatment effects will be calculated with 2-sided 95% CIs.

### Interim analysis

After 176 events, ie, 50% of the planned events, an interim analysis with the possibility to stop for efficacy or futility will be conducted. The significance level for a stop for efficacy is determined by an O’Brien and Fleming type α-spending function at overall 1-sided significance level of 2.5%. For futility stopping, a non-binding boundary (*P* ≥ .5) is defined. The sample size re-calculation will be based on conditional power using the treatment effect derived by the interim analysis.^19^

### Biomarker and imaging substudies

For a subgroup of patients, blood-based biomarkers will be studied in an accompanying scientific program to determine the association of cardiac and other biomarkers with cardiovascular outcome events and risk of recurrent AF in patients randomised to early rhythm control. Biomarkers will be measured at baseline and during follow-up at 24 months. For a subgroup of patients, brain imaging using MRI will be performed to study brain volume, morphology and vascular lesions at baseline and during follow-up at 24 months. Both baseline findings and changes occurring over time (eg, brain atrophy and silent new vascular lesions) will be studied in relation to treatment, cardiac rhythm throughout the trial, cognitive function and as surrogate imaging outcomes.

## Discussion

EAST-STROKE is an international, investigator-initiated, randomised controlled trial designed to assess the efficacy and safety of early rhythm control in patients with acute ischaemic stroke and AF. Despite the well-established role of OAC in preventing stroke in AF patients, the risk of recurrent stroke and major cardiovascular events remains substantial, even with optimal OAC. Given the high burden of AF-related strokes and their severe consequences, additional preventive strategies are needed.

Previous studies have suggested that maintaining sinus rhythm may offer further protection beyond OAC alone. However, prior rhythm-control trials focused on broader cardiology populations, and their applicability to acute stroke patients remains unclear. EAST-AFNET 4 demonstrated a significant reduction in cardiovascular events with early rhythm control in patients with recently diagnosed AF and cardiovascular comorbidities, but it did not specifically include patients with an acute ischaemic stroke.^9^ Subgroup analyses from EAST-AFNET 4,^17^ observational studies^11^ and a small pilot study^10^ indicate a potential benefit of rhythm control in stroke populations, supporting the hypothesis of EAST-STROKE. However, these findings require confirmation in a sufficiently powered trial.

EAST-STROKE will determine whether early rhythm control, in addition to usual care, reduces recurrent stroke and major cardiovascular events in patients with AF and acute ischaemic stroke. The trial incorporates a composite primary outcome, including recurrent ischaemic stroke, haemorrhagic stroke, unclassified stroke, cardiovascular death or hospitalisation due to heart failure or due to acute coronary syndrome, reflecting the broad spectrum of cardiovascular risks in this population.

EAST-STROKE is a therapy strategy trial delivering an innovative treatment concept, namely early rhythm control, using approved pharmacological and interventional therapies. The study’s adaptive design, including an interim analysis, permits an early stop for efficacy or futility. As part of the interim analysis, information from the current study population can be used to re-estimate the sample size more precisely, ensuring an adequate power.

Usual care in EAST-STROKE allows adaptation of acute stroke and AF management to new evidence emerging during the trial period in both study groups, thereby enhancing external validity and ensuring relevance to contemporary clinical practice.

Beyond clinical efficacy and safety, EAST-STROKE will assess functional outcomes, patient-reported health-related quality of life and cost-effectiveness. Given the disabling nature of recurrent strokes and the high healthcare burden associated with AF-related complications, understanding the broader impact of early rhythm control is crucial for guiding treatment decisions and healthcare policy. These clinical outcomes are complemented by brain MRI and blood biomarker substudies, enabling the exploration of novel risk markers, mechanistic pathways and additional clinically relevant outcomes.

## Summary and conclusions

EAST-STROKE is a therapy strategy trial delivering an innovative treatment concept, early rhythm control using approved therapies, in patients with acute ischaemic stroke and AF. The trial will provide evidence on whether early rhythm control, in addition to usual care, is safe and effective in preventing recurrent stroke and other major cardiovascular events compared to usual care alone. The EAST-STROKE trial addresses an unmet need and has the potential to improve outcomes for patients with AF and acute ischaemic stroke. Its findings will provide important evidence for clinicians, researchers and policymakers.
